# Prenatal multidisciplinary counseling for fetal congenital anomalies: A narrative review

**DOI:** 10.1002/ijgo.16068

**Published:** 2024-12-11

**Authors:** Licia Lugli, Cecilia Rossi, Alberto Berardi, Marisa Pugliese, Pier Luca Ceccarelli, Filomena Giulia Sileo, Giuseppe Chiossi, Giannina Contu, Olga Calabrese, Antonio La Marca, Emma Bertucci

**Affiliations:** ^1^ Neonatology Unit, Department of Medical and Surgical Sciences for Mothers, Children and Adults, University of Modena and Reggio Emilia Azienda Ospedaliero‐Universitaria Policlinico Modena Italy; ^2^ Psychology Unit, Department of Medical and Surgical Sciences for Mothers, Children and Adults, University of Modena and Reggio Emilia Azienda Ospedaliero‐Universitaria Policlinico Modena Italy; ^3^ Pediatric Surgery Unit, Department of Medical and Surgical Sciences for Mothers, Children and Adults, University of Modena and Reggio Emilia Azienda Ospedaliero‐Universitaria Policlinico Modena Italy; ^4^ Prenatal Medicine Center, Obstetrics and Gynecology Unit, Department of Medical and Surgical Sciences for Mothers, Children and Adults, University of Modena and Reggio Emilia Azienda Ospedaliero‐Universitaria Policlinico Modena Italy; ^5^ Genetic Unit, Department of Medical and Surgical Sciences for Mothers, Children and Adults, University of Modena and Reggio Emilia Azienda Ospedaliero‐Universitaria Policlinico Modena Italy; ^6^ Obstetrics and Gynecology Unit, Department of Medical and Surgical Sciences for Mothers, Children and Adults, University of Modena and Reggio Emilia Azienda Ospedaliero‐Universitaria Policlinico Modena Italy

**Keywords:** congenital anomalies, prenatal diagnosis, prenatal multidisciplinary counseling

## Abstract

**Introduction:**

Prenatal multidisciplinary counseling for fetuses with congenital anomalies involves a collaborative approach, integrating expertise from various medical fields.

**Aims and Approach:**

This comprehensive strategy aims to provide expectant parents with accurate information about the diagnosis, potential outcomes, and available interventions. Genetic counselors, obstetricians, neonatologists, and other specialists work together to address medical, psychological, and ethical aspects. The prenatal multidisciplinary counseling approach emphasizes open communication, fostering a supportive environment for the couple to express their concerns and ask questions. In the case of prenatally detected fetal congenital anomalies, several different scenarios can be delineated: (1) detection of surgically correctable congenital anomalies, (2) identification of genetic disease or fetal anomalies likely to result in disabilities, (3) discovery of severe and lethal congenital anomalies, and (4) encountering fetal anomalies that are not well‐defined, leading to an unclear scenario. The process of counseling includes discussing the possibility of pregnancy termination, treatment options, potential challenges, and emotional support, enabling expectant parents to make informed decisions aligned with their values and preferences. Additionally, the counseling process extends beyond the initial diagnosis, providing ongoing support as the pregnancy progresses and helping families to prepare for the difficulties they may face after the birth of the child with congenital anomalies. This collaborative effort not only focuses on the medical aspects but also considers the emotional and ethical dimensions of decision‐making.

**Conclusion:**

The multidisciplinary approach enhances the quality of care and empower parents, facilitating a more informed and compassionate journey throughout the prenatal period.

## Synopsis

This narrative review provides clinical practice directions on prenatal multidisciplinary counseling for the fetus with congenital anomalies, addressing medical, emotional, and ethical aspects.

## 1 INTRODUCTION

The incidence of congenital anomalies, also known as congenital disorders or birth defects, can vary based on factors such as geographical location, population demographics, and the specific type of anomaly.^1^, ^2^, ^3^, ^4^, ^5^ The WHO estimates that approximately 6% of live births worldwide are affected by congenital anomalies. Approximately 17%–42% of infant mortality can be attributed to congenital anomaly, with different rates among regions and countries.^2^ EUROCAT (European Surveillance of Congenital Anomalies) recorded a total prevalence of major congenital anomalies of 25 per 1000 births in 2021.^3^ Neural tube defects, heart defects, and chromosomal abnormalities are among the more frequently observed congenital anomalies.^1^, ^2^, ^3^, ^4^, ^5^ Advances in prenatal screening, including ultrasound examinations and maternal serum testing, have improved the ability to detect anomalies early in pregnancy. Invasive diagnostic procedures, such as chorionic villus sampling (CVS) and amniocentesis, provide more accurate information about the presence of congenital anomalies.^1^, ^6^, ^7^, ^8^, ^9^ The prenatal ultrasound detection rate is extremely variable (ranging from 10% to 90%), with differences depending on gestational age, body mass index of the patient, skills and experience of the sonographers, technical ultrasound machine, and type of congenital anomaly.^6^, ^7^, ^8^, ^9^ The RADIUS study compared the accuracy of ultrasonographic examinations performed in reference centers with those done in basic healthcare centers, reporting a detection rate for fetal anomalies of 35% in the ultrasound‐screening group in tertiary settings compared with 10% in the control group.^7^ Several studies have evaluated the effectiveness of ultrasound screening programs in detecting fetal anomalies in antenatal time.^1^, ^6^, ^7^, ^8^, ^9^ In a previous single‐center ultrasound screening for fetal anomalies, we found a sensibility of 87.5% and a specificity of 99.1% in prenatal congenital malformation detection.^8^ Prenatal diagnosis of congenital anomalies can have deep emotional and psychological impacts on the expectant parents. Comprehensive counseling and support services are crucial to help parents cope with the emotional challenges and make informed decisions.^1^, ^8^ A recent systematic review on counseling for fetal congenital anomalies included 24 articles and reported results of retrospective surveys. The quality of included studies was variable and only three studies assessed parental anxiety, reporting a significant decrease in anxiety following prenatal multidisciplinary counseling (PMC). Parents expressed a preference for counseling on all aspects of their baby's anomaly as soon as possible after prenatal diagnosis, and desired written, visual, and web‐based information resources, and support group contacts.^10^

Upon detection of a fetal structural anomaly, it is important to offer the pregnant woman an immediate consultation with a maternal‐fetal medicine specialist and a medical geneticist. Patients should be informed that prenatal ultrasound at 18–20 weeks can detect major structural anomalies in approximately 30%–60% of such cases.^1^, ^10^, ^11^ Suspected or confirmed fetal structural anomalies warrant an immediate referral to a tertiary ultrasound unit to optimize therapeutic strategies. PMC aims to discuss diagnostic tests, possible outcomes, treatment options, and emotional aspects, helping parents make informed decisions about their pregnancy. This communication helps to establish realistic expectations and facilitates a collaborative approach in providing optimal care for both the newborn and the family.^1^, ^10^, ^11^, ^12^, ^13^

### 1.1 PMC for congenital anomalies

Prenatal multidisciplinary counseling for congenital anomalies is typically performed by healthcare professionals and specialists in the field (Tables 1 and 2). The PMC should be unbiased and respectful of the patient's choice, culture, religion, and beliefs. The timing of prenatal counseling for congenital anomalies can vary based on several factors, including the nature of the anomaly, the gestational age at which it is detected, and the preferences of the expectant parents. Ideally, prenatal counseling begins as early as the first trimester and continues throughout the pregnancy. However, it becomes especially crucial when anomalies are suspected or detected during routine screenings or diagnostic tests. Early counseling allows parents to receive timely information about the nature of the anomaly, potential implications, and available options.^1^, ^10^, ^11^, ^12^, ^13^

**TABLE 1:** Timing and location of prenatal counseling.

Timing	1. *Early detection*: In cases where a congenital anomaly is detected early in pregnancy through routine ultrasound or specialized diagnostic tests, prenatal counseling can be initiated soon after the diagnosis. 3. *Multiple sessions*: Prenatal counseling may involve multiple sessions to address various aspects of the anomaly, allowing parents to digest information gradually and ask questions as they arise. 4. *Programmed later*: In some cases, counseling may be programmed for a later gestational age, especially if the anomaly is not detected until a later stage of pregnancy. The timing may also be influenced by the complexity of the anomaly and the need for additional diagnostic tests or consultations. 5. *Reassessment as needed*: Prenatal counseling should be an ongoing process, and the timing may be reassessed as needed. As the pregnancy progresses, additional medical information may become available, influencing decisions and the need for further counseling. 7. *Respecting parental preferences*: Healthcare providers should consider the preferences of the expectant parents when determining the timing of prenatal counseling. Some parents may prefer to receive information early, while others may choose to delay counseling until they feel emotionally prepared.
Location	1. *Reserved space*: Counseling should take place in a quiet and private environment, away from external distractions, to promote privacy and the sincerity of the conversation. 2. *Comfort and welcoming atmosphere*: The decor and atmosphere of the room should be welcoming and comfortable, contributing to putting the couple at ease during the counseling session. 3. *Confidentiality*: Ensure that the location respects the couple's confidentiality. Confidentiality is crucial to create an environment where they feel free to express concerns and emotions without fear. 4. *Possibility to stay after*: If the couple wishes to stay in the same space after counseling to reflect or discuss further, it should be possible to do so discreetly and without time pressures. 5. *Accessibility*: Verify that the location is easily accessible for the couple, ensuring there are no obstacles that could create discomfort. 6. *Additional resources*: In the counseling space, additional resources such as informational material, books, or online resources might be available to support the couple in their decisions and journey. 7. *Cultural sensitivity*: Consider cultural sensitivity in designing the space, respecting the diverse cultural and religious needs of the individuals involved. 8. *Involvement of partners*: Include the partner in the design of the space, if possible, to ensure that their comfort and well‐being are also taken into consideration.

**TABLE 2:** Who and how to perform prenatal counseling.

Who performs counseling	1. *Genetists*: They play a crucial role in explaining genetic information, discussing the implications of genetic conditions, and providing support to individuals and families. 2. *Maternal‐fetal medicine specialists (perinatologists)*: Maternal‐fetal medicine specialists are obstetricians with additional training in managing high‐risk pregnancies. They may provide medical information and guidance related to congenital anomalies during pregnancy. 3. *Obstetricians/gynecologists (OB/GYNs)*: They are often involved in prenatal care and can provide information and counseling regarding congenital anomalies. 4. *Neonatologists*: They may offer insights into the potential medical interventions and care needed for infants with congenital anomalies. 6. *Nurses*: Nurses may provide support, education, and information to expectant parents, assisting in understanding the medical aspects of congenital anomalies. 8. *Psychologists*: Mental health professionals, such as psychologists, may be involved in offering emotional support and counseling to parents dealing with the psychological impact of a prenatal diagnosis. 9. *Ethicists*: In some cases, ethicists may be consulted to help navigate complex ethical considerations that may arise in the context of congenital anomalies. 10. *Interdisciplinary teams*: Prenatal counseling for congenital anomalies often involves an interdisciplinary team that collaborates to address various aspects of care, including medical, genetic, psychosocial, and ethical considerations.
How to perform counseling	1. *Acknowledge emotions*: Recognize and validate the parents' fears and concerns. 2. *Transparent communication*: Provide clear and honest information about the fetal malformation, potential outcomes, and available options. 3. *Create a supportive environment*: Foster a supportive and non‐judgmental atmosphere where parents feel comfortable expressing their emotions. 4. *Offer to answer questions*: Invite parents to ask questions and address any uncertainties they may have. Providing accurate information can help alleviate some of their fears. 5. *Emphasize ongoing support*: Assure parents that the counseling and support will continue throughout the process, from prenatal care to postnatal care. 6. *Discuss possibilities for future interactions*: Explore options for parents to see and bond with their baby, even if it's through imaging techniques or ultrasounds. 7. *Involve other supportive professionals*: Collaborate with social workers, psychologists, or counselors who specialize in perinatal care to provide additional emotional support and coping strategies. 8. *Respect cultural and religious beliefs*: Be mindful of the cultural and religious background of the parents. 9. *Coordinate with maternity services*: Involve maternity services to discuss birthing options and postnatal care plans, contributing to a comprehensive approach that considers the entire perinatal journey.

The ability of first‐trimester ultrasound screening to detect different malformations is variable. A recent systematic review concluded that the use of a standardized anatomic protocol was the most crucial factor to improve the sensitivity of first‐trimester ultrasound screening for anomalies.^10^, ^11^, ^12^, ^13^ It is therefore recommended that screening for severe structural abnormalities should be extended to the first trimester. The detection rate for first‐trimester screening was 43.1% in the study by Liao et al.^12^ A robust high detection rate for anencephaly, exencephaly, cephalocele, holoprosencephaly, exomphalos, gastroschisis, pentalogy of Cantrell, sirenomelia, and body stalk anomaly was achieved during routine first‐trimester scans.^12^, ^13^ Most of these fetal anomalies detected in the first trimester have adverse prognosis and often result in termination of the pregnancy. In cases of termination of pregnancy, stillbirth, or neonatal death, healthcare professionals should advocate for a comprehensive autopsy conducted by a perinatal or pediatric pathologist to yield crucial insights into the diagnosis and etiology of structural fetal anomalies. Otherwise, in ongoing pregnancies with fetal structural anomalies, ultrasound examination should be repeated (with frequency tailored to the specific anomaly) to monitor the progression of the anomaly and potentially uncover additional anomalies. This ongoing evaluation is vital for informed counseling and obstetrical or perinatal management decisions. Once a fetal structural anomaly is identified by two‐dimensional (2D) ultrasound, complementary imaging techniques such as fetal echocardiography, neurosonography, 3D obstetrical ultrasound, and fetal magnetic resonance imaging (MRI) may offer valuable insights, particularly in complex cases. Furthermore, parental blood testing and invasive prenatal procedures might be necessary to clarify the diagnosis for fetuses presenting with isolated or multiple structural anomalies. Parents should be informed that major or minor fetal structural anomalies, whether isolated or multiple, may be part of a genetic syndrome, despite a normal fetal karyotype.^10^, ^11^, ^12^, ^13^, ^14^ Prenatal chromosomal microarray analysis (CMA) is increasingly used in prenatal diagnosis and may add 8% to the diagnostic yield compared with conventional karyotyping for fetuses with ultrasound anomalies. CMA was confirmed to be useful in the patient's decision‐making process regarding whether to continue the pregnancy, not only after a positive result. In fact, a negative result also helped parents' choices, often being reassuring, especially in the case of an isolated fetal anomaly with a good prognosis. In fetuses with major anomalies or multiple organ system anomalies with a non‐diagnostic CMA, exome sequencing is indicated. The diagnostic yield of the exome ranged from a low of 6% to as high as 80%. The challenge of exome sequencing in prenatal diagnosis remains the need for a qualified genetic counselor to interpret the results and the duration of the test, which must be short for the duration of the pregnancy.^14^, ^15^

Prenatal multidisciplinary counseling should be an ongoing process, and the timing may be reassessed as needed. Early initiation of counseling provides an opportunity for psychosocial support, addressing the emotional needs of the parents as they navigate the challenges associated with a prenatal diagnosis. In some cases, PMC may be programmed for a later gestational age, especially if the anomaly is not detected until a later stage of pregnancy. Women should receive information regarding the abnormal ultrasound findings in a clear, sympathetic, timely fashion, and in a supportive environment that ensures privacy. Referral to the appropriate pediatric or surgical subspecialists should be considered to provide the most accurate information, concerning the anomalies and the associated prognosis.

Prenatal multidisciplinary counseling can occur in various settings, including the obstetrician's office, maternal‐fetal medicine clinics, or specialized genetic counseling centers. It may also take place in hospitals with neonatologists and other specialists. Counseling should take place in a quiet and private environment, away from external distractions, to promote privacy and the sincerity of the conversation. The decor and atmosphere of the room should be welcoming and comfortable, contributing to putting the couple at ease during the counseling session. Ensure that the location respects the couple's confidentiality. Confidentiality is crucial to create an environment where they feel free to express concerns and emotions without fear. If the couple wishes to stay alone in the same space after counseling to reflect or discuss further, it should be possible to do so discreetly and without time pressures. Prenatal counseling is a delicate and personal moment, and creating an appropriate environment is essential to facilitate open communication and the well‐being of the couple (Tables 1 and 2).

Prenatal multidisciplinary counseling for congenital anomalies is typically provided by a team of healthcare professionals with expertise in various aspects of medical, genetic, and psychosocial care. The composition of the counseling team can depend on factors such as the nature of the anomaly, the stage of pregnancy, and the specific needs and preferences of the parents. (Tables 1 and 2). Obstetricians and maternal‐fetal medicine specialists often initiate discussions about potential anomalies. Genetic counselors play a crucial role in assessing hereditary risks. In cases of confirmed anomalies, neonatologists and pediatric specialists should be involved to discuss postnatal care. The collaborative effort of healthcare professionals ensures comprehensive counseling that covers medical information, emotional support, and decision‐making for expectant parents facing the challenges of congenital anomalies.^1^, ^10^, ^11^, ^16^, ^17^ The use of drawings or figures to depict the fetal anomalies can help the couple to get a more concrete idea of the situation and better understand it.

If early or urgent postnatal management may be required, delivery at a center that can provide the appropriate neonatal care should be warranted. When any congenital structural anomaly has been identified prenatally, a comprehensive newborn assessment is essential for diagnosis and counseling on the etiology, prognosis, and recurrence risk for future pregnancies, especially when the etiology has not been clearly identified prenatally.^10^, ^11^, ^12^, ^13^, ^14^, ^15^, ^16^, ^17^

**FIGURE 1:**
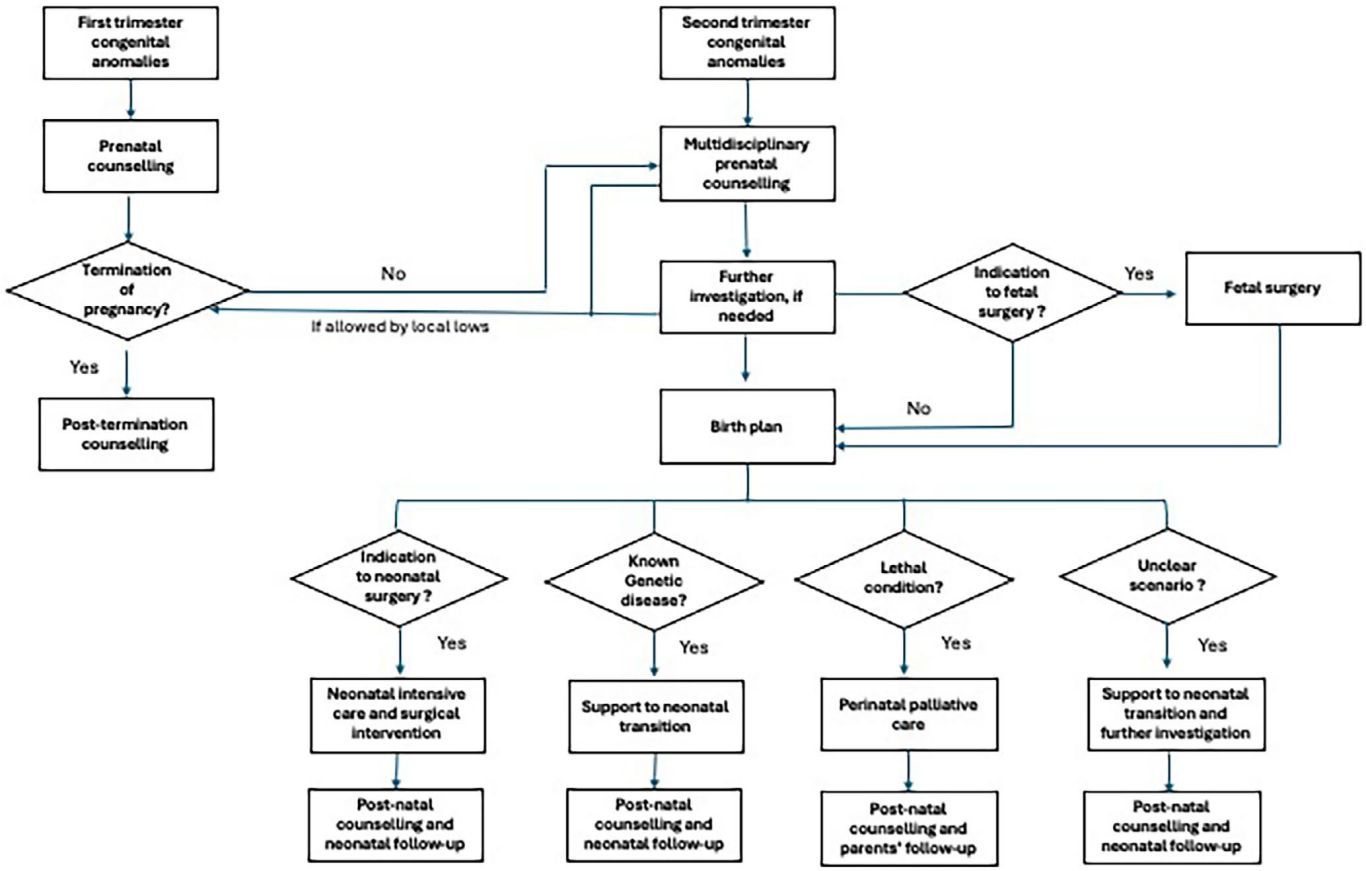
Prenatal counseling flowchart.

Based on literature data and our personal experience, in the case of prenatally detected fetal congenital anomalies, several different scenarios can be delineated: (1) detection of surgically correctable congenital anomalies, (2) identification of fetal anomalies likely to result in disabilities, (3) discovery of severe and lethal congenital anomalies, and (4) encountering fetal anomalies that are not well‐defined, leading to an unclear scenario. These different situations are described in the following paragraphs and showed in the prenatal counseling flow‐chart (Figure 1).^10^, ^11^, ^12^, ^13^, ^14^, ^15^, ^16^, ^17^

### 1.2 PMC for surgically correctable congenital anomalies

**FIGURE 2:**
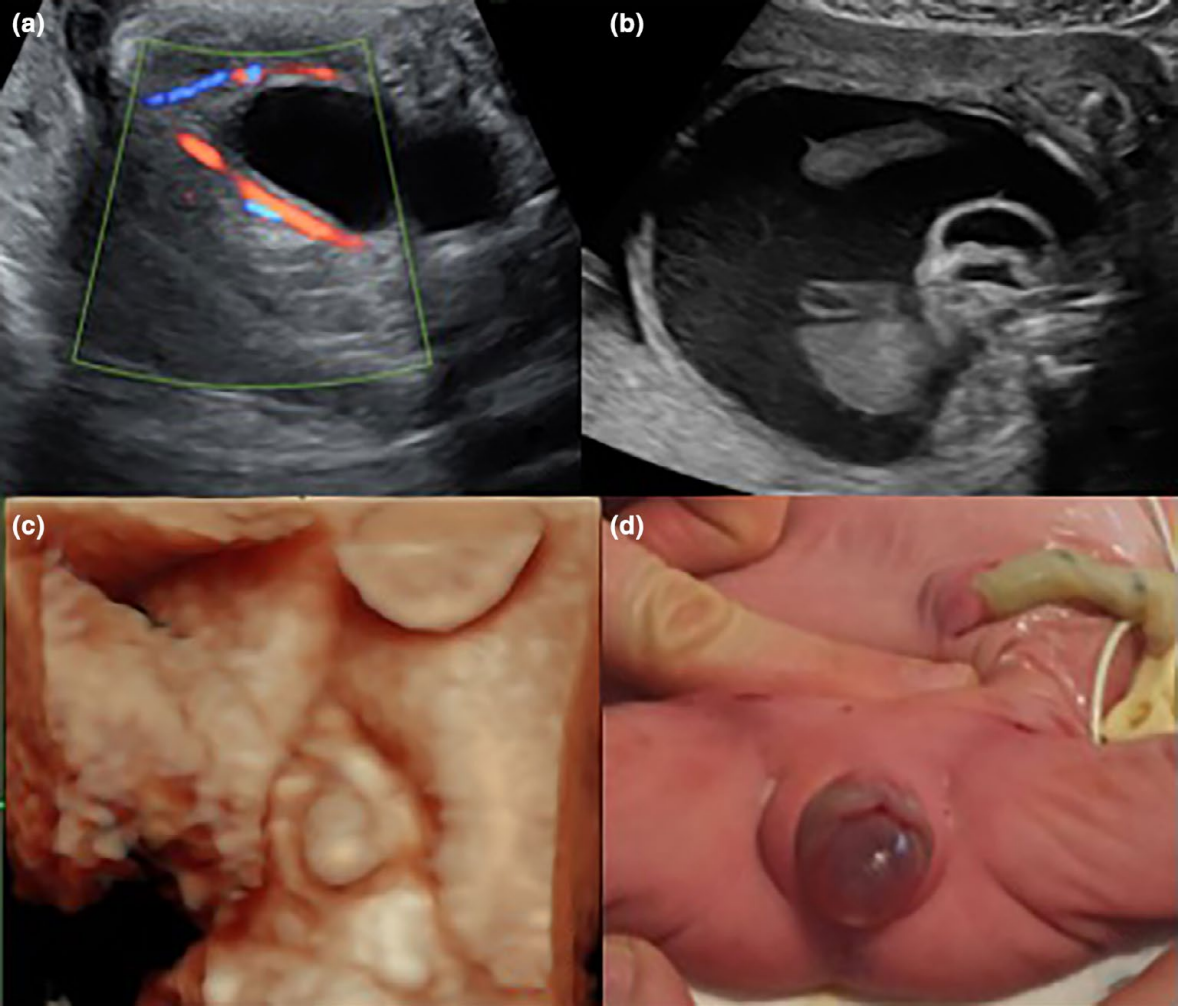
Imperforate hymen with hydrometrocolpos and fetal ascites. (a–c) Prenatal two‐dimensional (2D) (a, b) and 3D (c) ultrasound imaging. (d) Imperforate hymen in the newborn, later treated surgically.

**FIGURE 3:**
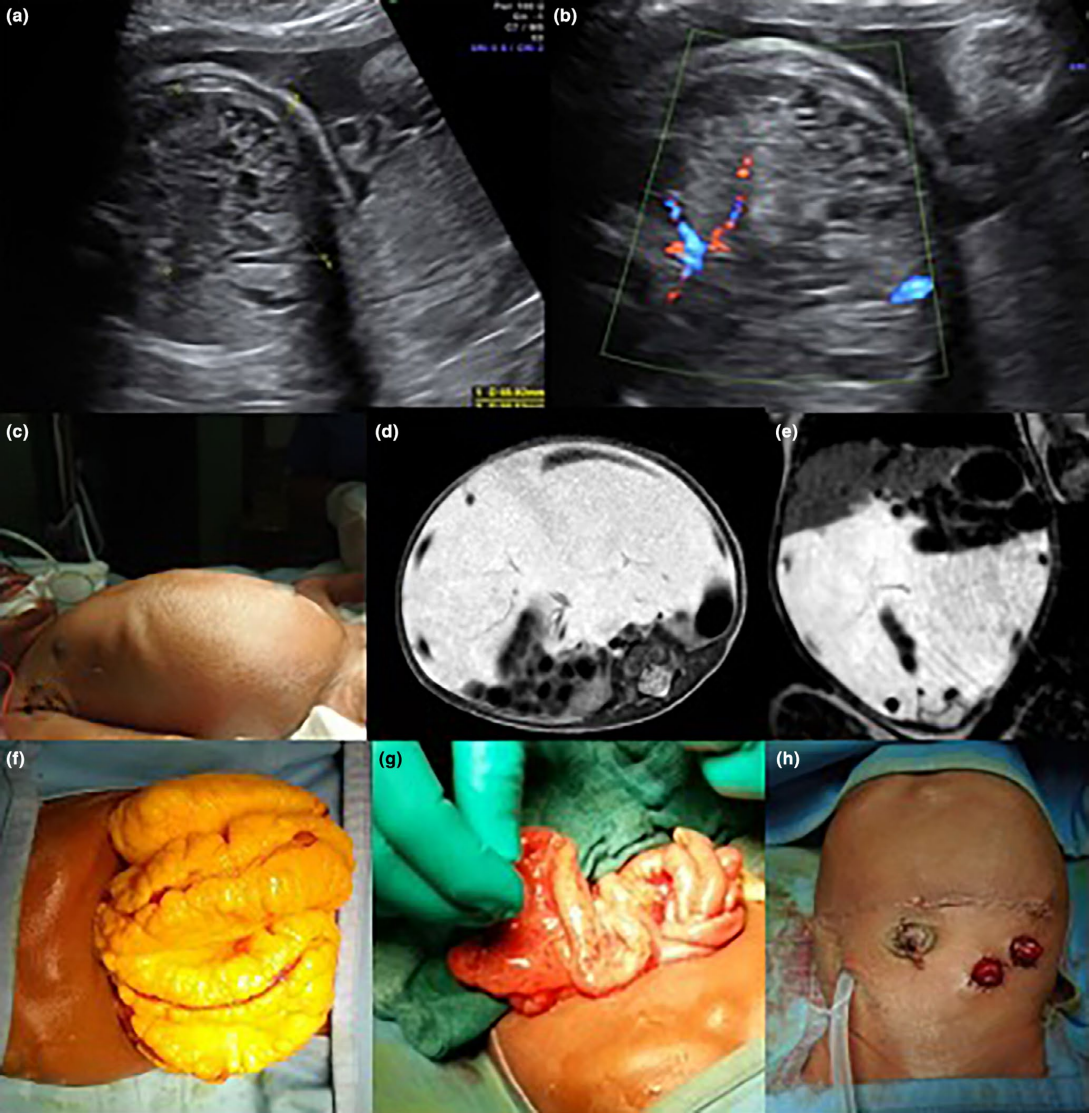
Prenatal diagnosis of a large abdominal mass identified as a lymphangioma, treated surgically. (a, b) Prenatal ultrasound showing an abdominal mass, suspected to be a lymphangioma. (c) Abdominal distension in the newborn at birth. (d, e) Postnatal abdominal magnetic resonance imaging confirms the prenatal suspicion of lymphangioma. (f–h) Laparotomy reveals an extensive intestinal lymphangioma, which is drained and removed with the creation of intestinal stomas.

Several fetal anomalies are surgically correctable, depending on the nature and severity of the condition (Table 3).^1^, ^17^ It is important to note that the decision to pursue surgical correction depends on factors such as the specific diagnosis and the overall health of the fetus. A multidisciplinary team of healthcare professionals, including pediatric surgeons, neonatologists, and genetic counselors, typically collaborates to assess and plan the appropriate interventions for each individual case. Expertise in the surgical correction of congenital malformations can significantly impact the perinatal management of prenatally diagnosed anomalies.^10^, ^17^ Previous research has underscored the beneficial effects of prenatal surgical consultation, with findings indicating that it influenced the site of delivery in 48% of cases, altered the mode of delivery by 10%, led to a decision to terminate a pregnancy in 4.6% of cases, and resulted in a change of diagnosis in 7% of cases.^18^ The advent of prenatal diagnosis has substantially enhanced our comprehension of surgically remediable congenital malformations. Standard ultrasound screening typically conducted at 18–20 weeks' gestation detects anomalies, categorizing these pregnancies as high risk. Further invasive diagnostic procedures like amniocentesis or CVS may be offered to high‐risk pregnancies. Structural abnormalities that are challenging to delineate via ultrasound, such as hindbrain lesions or cases of oligohydramnios, can be better visualized through MRI. With the increasing range of options and sophistication of diagnostic methods, parents today are faced with more information, choices, and decisions than ever before, which can create as well as solve dilemmas. The ideal purpose of prenatal diagnosis and testing is to achieve 100% accuracy without fetal loss or injury and with no maternal risk. Essential to PMC is the understanding of the specific surgical condition's prenatal natural history, the limitations of prenatal diagnosis, and the detection of associated anomalies. The parents receive information about the nature of congenital anomaly, timing of the surgery, the recommended surgical procedure, potential risks and benefits, the medical team involved, and the expected outcomes post‐surgery (Figures 2 and 3). In some cases, fetal surgical procedures may be indicated. The risks and indications of fetal intervention programs should be fully understood by parents. Developmental malformations amenable to fetal intervention can be usefully categorized into five evidence‐based groups of conditions that benefit from fetal therapy. These range from level I, where conditions are associated with significant mortality or very severe morbidity if left untreated (such as twin‐to‐twin transfusion syndrome, myelomeningocele, and congenital diaphragmatic hernia), to level V, where conditions for which the use of fetal therapy is still controversial, such as osteogenesis imperfecta and aqueductal stenosis. Fetal therapy must meet the following three criteria to be ethically acceptable:

1. It should be life‐saving or prevent or substantially alleviate serious or irreversible disease, injury, or disability in the fetus.
2. The proposed therapy should have a low risk of fetal death and a low or manageable risk of serious disease, injury, or handicap to the fetus.
3. The risk of mortality and morbidity (illness, injury, or disability) to the mother should be very low.

**TABLE 3:** Some surgically correctable malformations are reported.

Malformation	Discussion
Congenital heart defects	Many structural heart abnormalities can be corrected through surgical interventions, such as atrial septal defect (ASD) repair, ventricular septal defect (VSD) repair, or complex heart surgeries for more intricate conditions
Neural tube defects	Conditions like spina bifida may require surgical correction to close the defect and prevent further damage to the spinal cord
Abdominal wall defects	Conditions like gastroschisis or omphalocele can often be corrected through surgical procedures to place the organs back into the abdominal cavity and close the opening
Esophageal atresia and tracheoesophageal fistula	Anomalies affecting the development of the esophagus can be surgically corrected to establish proper continuity and function
Gastrointestinal anomalies	Certain gastrointestinal anomalies, like imperforate anus or intestinal atresia, require surgical correction to restore normal bowel function
Cleft lip and palate	Surgical procedures can correct cleft lip and palate, improving facial aesthetics and ensuring proper functioning of the mouth and palate
Urinary tract anomalies	Conditions such as hydronephrosis or obstructive uropathy may require surgical interventions to correct anatomical issues and restore normal urinary function
Limb anomalies	Some limb abnormalities, such as polydactyly or syndactyly, can be surgically corrected to improve function and appearance
Diaphragmatic hernia (CDH)	Lung hypoplasia and pulmonary hypertension account for most morbidity and mortality in CDH. Associated anomalies (30%–40%) signify a grave prognosis with a reduced survival rate

Otherwise, in case the baby requires specialized care immediately after birth, neonatologists are involved to ensure a seamless transition from prenatal to postnatal medical management. The type of surgical intervention depends on the congenital anomaly identified. In some cases, the surgical procedure may be complex, and the newborn may require intensive care support. Therefore, the prognosis remains uncertain during prenatal life. In any case, parents can be educated about postnatal care, potential complications, and long‐term outcomes. A plan is established for follow‐up appointments and continued monitoring of the baby's health. Collaboration among healthcare professionals is fundamental to optimize outcomes for both the baby and the parents.^17^, ^18^, ^19^

### 1.3 PMC for fetal congenital anomalies leading to disabilities

The prenatal diagnosis of a genetic condition carries important implications, as it is often associated with a situation of chronic disease in the postnatal period. Moreover, certain genetic diseases and chronic conditions diagnosed prenatally can lead to disabilities in the child.^10^, ^16^, ^17^, ^20^ Disabilities resulting from prenatal diagnoses may include intellectual disabilities, epilepsy, developmental delays, physical impairments, sensory impairments (such as blindness or deafness), and neurodevelopmental disorders. Fetal medicine involves testing and screening procedures performed during pregnancy to detect the presence of genetic abnormalities or other conditions in the developing fetus, including non‐invasive prenatal testing (NIPT) using cell‐free fetal DNA in the maternal bloodstream and invasive procedures like CVS and amniocentesis. Fetal echocardiography, neurosonography and prenatal MRI may be indicated. PMC for conditions leading to disabilities is a multifaceted process that requires a comprehensive understanding of medical, ethical, and psychosocial aspects. As advancements continue, the goal remains to empower expectant parents with accurate information, empathy, and support to make informed decisions about their pregnancy. The first step in effective prenatal counseling involves accurate identification and diagnosis of conditions that may lead to disabilities. Detailed discussions focus on the nature of the anomaly, potential functional limitations, and available interventions and treatments. In this contest, PMC also involves navigating complex ethical dilemmas surrounding decisions such as continuation or termination of pregnancy, considering the perspectives of healthcare professionals, parents, and the broader societal context. Additionally, the psychosocial impact of prenatal counseling on expectant parents is a critical aspect.^10^, ^16^, ^17^, ^20^

Emotional aspects are addressed, providing support for parents as they process the information and prepare for the unique parenting journey ahead. Emphasis is placed on the child's potential and the importance of fostering a positive environment. It is also fundamental to plan the postnatal follow‐up, to continue diagnostic investigation, to detect early sign of impairment and to direct the child to rehabilitative intervention in a timely manner. Plans for early intervention and rehabilitation services should be discussed, aiming to optimize the child's development and functional abilities.^10^, ^16^, ^17^, ^20^

### 1.4 PMC for lethal congenital anomalies

**FIGURE 4:**
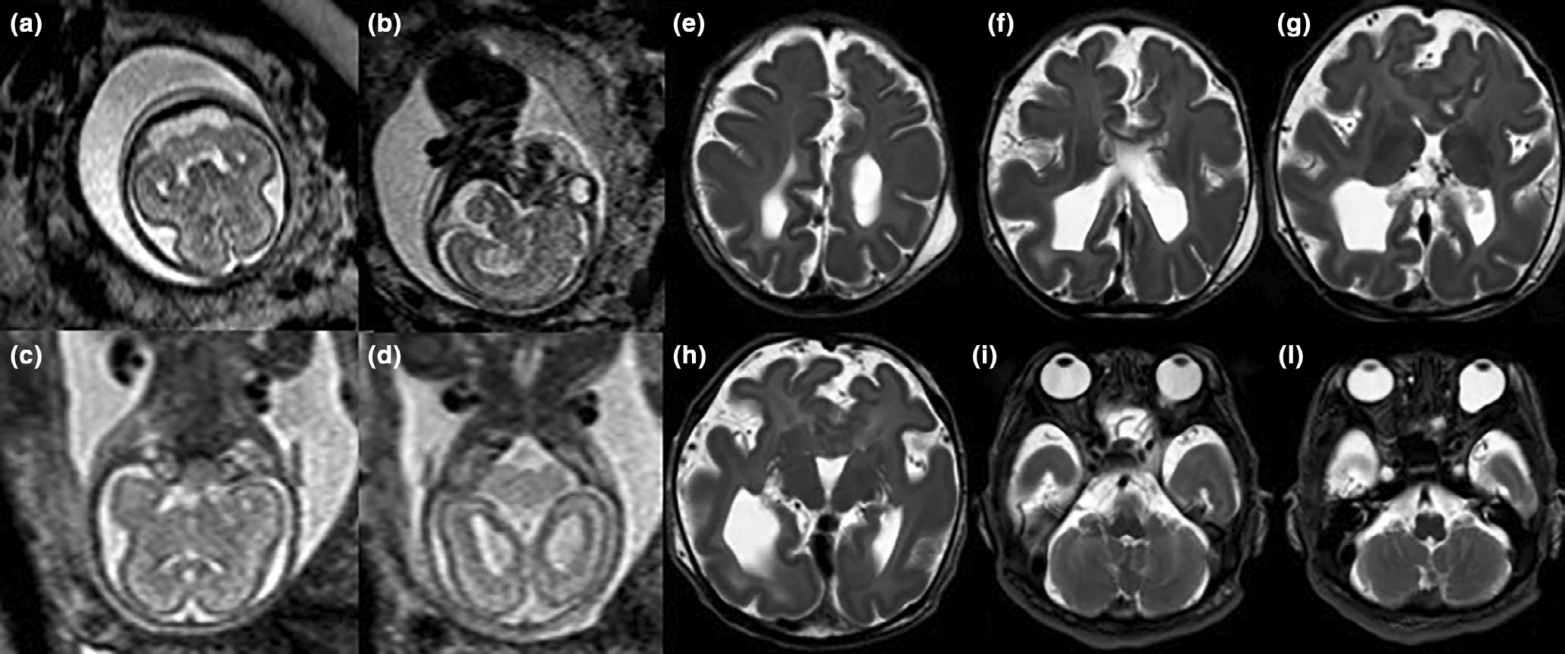
Prenatal diagnosis of lobar holoprosoencephaly. The baby was born alive but developed intractable epilepsy and died in a few days. (a–l) Fetal magnetic resonance imaging (MRI) (20 weeks of gestational age) (a–d) and postnatal cerebral MRI (e–l) showed absent anterior third of the corpus callosum, absent pellucid septum, unseparated frontal lobes, diffuse irregular and abnormal cortical profile, biparietal and fronto‐occipital cerebral diameter reduction.

In a hypothetical scenario involving congenital lethal anomalies, a comprehensive ultrasound reveals severe congenital anomalies that are incompatible with life (Table 4; Figure 4).^1^, ^21^, ^22^, ^23^, ^24^, ^25^ The anomalies are identified in multiple organ systems, and the prognosis is deemed incompatible with long‐term survival.

**TABLE 4:** Severe fetal congenital anomalies often described as “lethal”.

Severe congenital anomalies	Prevalence	Probability of live birth (in absence of termination of pregnancy)
Renal agenesis	17/10000	Not reported
Anencephaly	10/10000 pregnancies or 2.6/10000 births	62%–72%
Thanatophoric dysplasia	0.4/10000	Not reported
Trisomy 18	2.6/10000	48%–51%
Trisomy 13	1.2/10000	28%–46%
Holoprosencephaly	0.5/10000	3%

Prenatal counseling is initiated promptly after the detection of the lethal anomalies, respecting the emotional and psychological impact on the parents. The counseling sessions occur in a compassionate and supportive environment, typically in a hospital setting with specialized perinatal and palliative care teams. Healthcare professionals, including obstetricians, neonatologists, genetic counselors, and palliative care specialists, provide detailed information about the nature of the anomalies and the expected prognosis. Emphasis is placed on the fact that the condition is not survivable. The focus is on providing emotional support, understanding, and empathy to the parents as they navigate the difficult decisions ahead. Counseling addresses grief, coping mechanisms, and the range of emotions associated with this challenging situation. The parents are guided through the process of decision‐making, which may involve choices regarding continuing the pregnancy, considering palliative care, or exploring options for a supportive delivery plan. If the decision is made to continue the pregnancy, discussions include planning for compassionate end‐of‐life care, ensuring the baby's comfort and minimizing suffering. This hypothetical scenario underscores the importance of sensitivity, open communication, and comprehensive support when facing the challenging reality of congenital lethal anomalies.^21^, ^22^, ^23^, ^24^, ^25^

### 1.5 PMC in the case of unclear scenario

**FIGURE 5:**
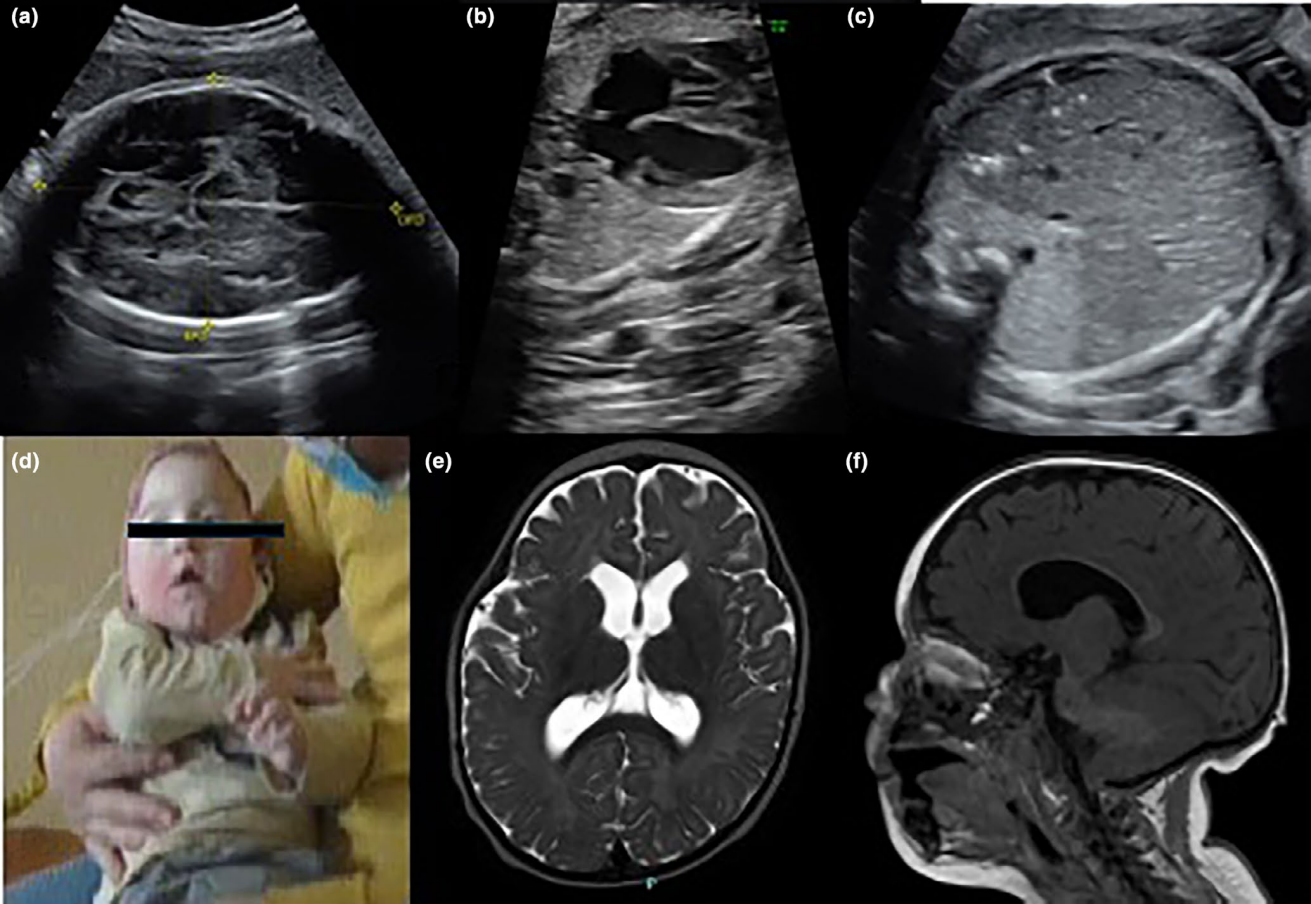
Prenatal unclear scenario in a case with a postnatal diagnosis of Arboleda–Tham syndrome, a recently described genetic disease due to *KAT6A* pathogenic variant and associated with intellectual disability. (a–c) Prenatal ultrasound (31 weeks of gestation) showed an abnormal cranial shape with dolichocephaly (a), abnormal cardiac axis (b), and liver calcifications (c). (d) The patient at 1 year of life showing microcephaly and severe global delay. (e, f) Cerebral magnetic resonance image (10 months of age) showing supratentorial ventriculomegaly, reduced white matter, thin corpus callosum, and tonsillar descent (Arnold–Chiari type 1 malformation).

If prenatal imaging reveals anomalies that are not immediately identifiable or fall into a gray area of uncertainty, a second opinion is desirable to better define the scenario. Genetic ascertainments or MRI evaluation may be indicated. In some cases, despite further assessments, the nature and severity of the anomalies remain still unclear (Figure 5). Prenatal counseling begins after the detection of the unclear anomalies to address concerns and provide information, even if a definitive diagnosis is not yet available. Counseling sessions occur in a collaborative setting involving obstetricians, neonatologists, genetic counselors, and specialists who can further investigate and interpret the anomalies. Additional diagnostic tests may be recommended: further specialized tests, such as advanced imaging or genetic testing, may be proposed to gain a clearer understanding of the anomalies.^1^, ^10^, ^11^, ^17^, ^20^ These tests help to refine the diagnosis and provide more accurate information to guide counseling. Discussions focus on the current understanding of the anomalies, potential diagnostic pathways, and the implications of different outcomes. The uncertain nature of the situation is communicated transparently to the parents. Recognizing the emotional strain of uncertainty, counseling emphasizes emotional support, offering resources for coping and strategies for decision‐making once more information becomes available. As additional diagnostic results emerge, counseling sessions are revisited to provide updated information, discuss potential treatment options, and support parents in making decisions based on the new insights. Regular follow‐up appointments are scheduled to monitor the pregnancy, reassess anomalies as more information becomes available, and adjust counseling accordingly. This hypothetical scenario underscores the importance of ongoing communication, collaboration among healthcare professionals, and adaptive counseling strategies when facing situations where congenital anomalies are initially unclear.^10^, ^11^, ^12^, ^13^, ^14^, ^15^, ^16^, ^17^, ^18^, ^19^, ^20^ In this situation more than others, it is fundamental the evaluation of the newborn at birth, to establish the most appropriate treatment, proportionate to the baby's need.

## 2 DECISION TO TERMINATION OF PREGNANCY

**FIGURE 6:**
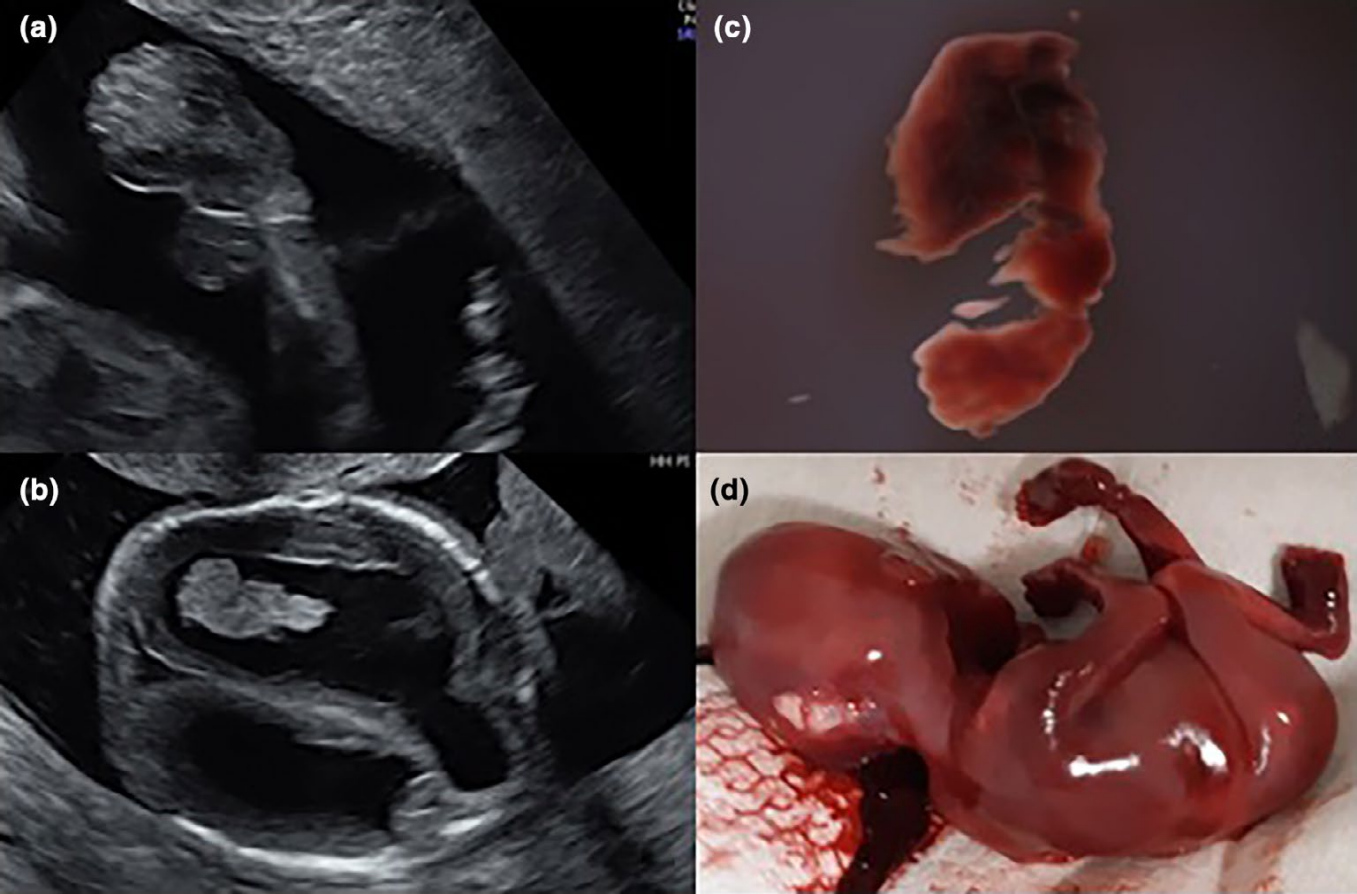
(a, b) Prenatal ultrasound scan in fetus with multiple malformations at 16 gestational weeks. (c, d) Therapeutic interruption due to posterior occipital encephalocele and lower limb defect from amniotic band syndrome.

The decision to terminate a pregnancy after counseling is a deeply personal one, influenced by various factors, including the nature of fetal anomalies, the parents' values, and medical considerations. The counseling process plays a crucial role in supporting individuals or couples through this difficult decision. Prenatal counseling provides comprehensive information about the nature and severity of congenital anomalies, potential outcomes, and available treatment options. This empowers parents to make informed decisions aligned with their values. Counseling involves discussing various options, including continuation of the pregnancy, palliative care, or, in some cases, termination (Figure 6). The healthcare provider addresses questions and concerns, fostering an open and non‐judgmental atmosphere. If termination is considered, the healthcare team discusses the medical aspects, including the procedure, potential risks, and emotional aspects involved.

Compassionate communication helps individuals navigate the emotional complexities associated with termination. Healthcare providers emphasize the autonomy of the individual or couple in making decisions about their pregnancy. Ensuring that individuals are aware of the legal and ethical aspects of termination, including relevant regulations in their region, is an integral part of counseling. Post‐procedure, continued counseling is offered to provide emotional support and address any psychological implications. Grief counseling and access to mental health professionals may be recommended. The healthcare team ensures ongoing follow‐up care, both physically and emotionally, to monitor the individual's well‐being and provide additional support as needed. Termination of pregnancy is a complex decision, and the counseling process aims to guide individuals through it with sensitivity, respect, and the necessary emotional support.^1^, ^11^, ^21^

When discussing pregnancy termination, cultural sensitivity is critical. In some cultures, there may be a strong belief in fate or divine will, leading to the acceptance of all aspects of life, including raising a child with a disability. For these families, the idea of terminating a pregnancy might be deeply challenging or even considered morally unacceptable. In other cultures, particularly those that are more focused on personal autonomy, decisions about whether to continue a pregnancy may be framed in terms of what is best for the family unit or the individual woman, which might include considering the potential for raising a child with special needs. Therefore, it is important to approach this discussion with care, understanding, and without imposing one's own values.

## 3 PALLIATIVE PERINATAL CARE

**FIGURE 7:**
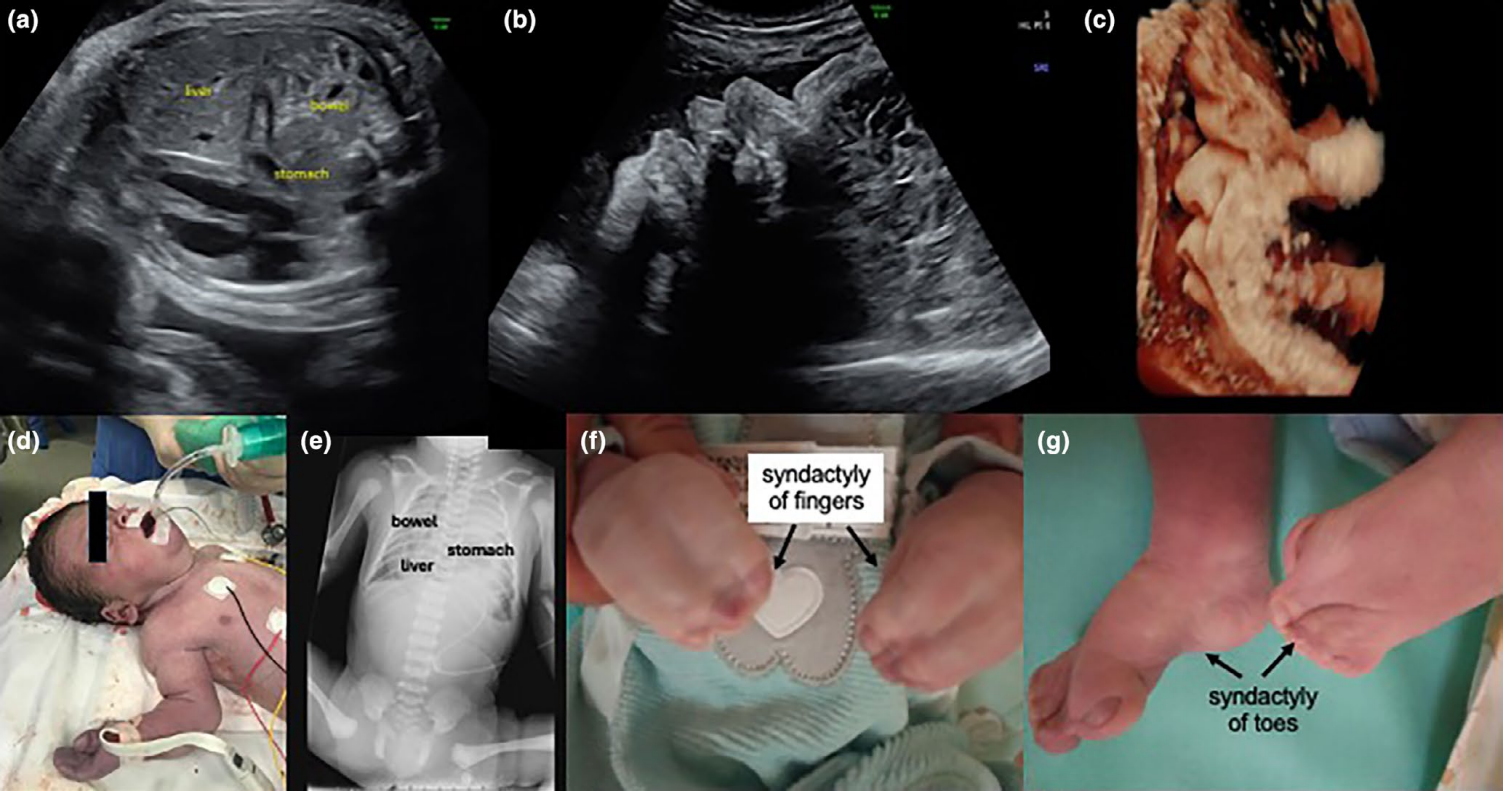
Prenatal diagnosis of a large diaphragmatic hernia and turricephaly in Apert syndrome. Prenatal counseling revealed the couple's desire to provide all possible care for the newborn, but due to the ineffectiveness of the treatments, palliative care was adopted soon after (withdrawing is equal to withholding). Afterward, the parents wrote a book about their son's story, *9 Months Lived Intensely*. (a) Prenatal scan showing stomach, bowel, and liver in the thorax. (b, c) Two‐dimensional (2D) and 3D prenatal scan showing dysmorphic features including macrocrania and frontal bossing. (d) The newborn after birth. (e) Chest X‐ray showing herniated bowel, stomach, and liver. (f, g) Syndactyly of the fingers and toes.

Perinatal palliative care is typically considered in situations where a fetus is diagnosed with a life‐limiting or life‐threatening condition, and the expected prognosis suggests that the baby's life may be short‐lived (Figure 7). Conditions that might prompt the consideration of perinatal palliative care are reported in Table 5.^11^, ^21^, ^22^, ^23^, ^24^, ^25^

Perinatal palliative care aims to provide comfort, support, and quality of life for the baby during the time they have, while also offering emotional support and guidance for the parents and family. It is a holistic approach that takes into consideration the physical, emotional, and spiritual well‐being of all involved. The decision to pursue perinatal palliative care is deeply personal and should involve open communication and collaboration between healthcare providers and parents. Prenatal counseling involves providing comprehensive information about the nature and severity of congenital anomalies. Counseling sessions focus on exploring various options, including continuation of the pregnancy with a palliative care approach. The healthcare team discusses what palliative care entails, addressing questions and concerns, and emphasizing the importance of comfort and quality of life for the baby. If palliative perinatal care is chosen, the healthcare provider outlines the medical aspects, potential interventions, and supportive measures to ensure the baby's comfort. Parents are informed about what to expect during the pregnancy, delivery, and postnatal period. Emotional support is a central component of counseling, recognizing the emotional toll of such decisions. Providers should offer empathy, sensitivity, and a safe space for parents to express their feelings and concerns.^11^, ^21^, ^22^, ^23^, ^24^, ^25^

**TABLE 5:** Life‐limiting or life‐threatening conditions that might prompt the consideration of perinatal palliative care.

Life‐limiting or life‐threatening condition	Examples
Severe congenital anomalies	Conditions affecting multiple organ systems or severe structural abnormalities where curative treatment may not be feasible
Genetic or chromosomal abnormalities	Conditions resulting from genetic or chromosomal abnormalities that significantly impact the baby's health and life expectancy
Neurological disorders	Severe neurological conditions, rapidly progressive or irreversible, that may lead to profound disabilities or significantly limit the baby's quality of life
Early‐onset, life‐limiting conditions without curative options	Any medical situation where the prognosis indicates a limited life expectancy, and there are no curative treatment options available

### 3.1 Creating a birth plan

If palliative perinatal care is chosen, discussions include creating a birth plan that aligns with the parents' wishes and provides a compassionate and supportive environment during labor and delivery. After delivery, continued counseling and support are essential. This includes addressing grief and loss, facilitating connections with bereavement services, and providing resources for ongoing emotional support. Recognizing and respecting cultural and spiritual beliefs are integral to the counseling process, tailoring support to the individual needs and preferences of the parents.

The counseling process aims to ensure that parents have the information and emotional support needed to make decisions that are in the best interests of both the baby and the family, considering the unique circumstances of congenital anomalies.^21^, ^22^, ^23^, ^24^, ^25^

## 4 CONCLUSION

In conclusion, this review demonstrates that prenatal counseling for congenital anomalies is a fundamental aspect of comprehensive perinatal care. It involves providing information, support, and guidance to parents facing the challenges associated with a diagnosed anomaly. The main limitation is that the paper likely relies on theoretical perspectives and existing literature, without including empirical studies or primary data that directly assess the effectiveness of prenatal counseling for congenital anomalies.

Early identification of congenital anomalies through prenatal screenings or diagnostic tests allows for timely counseling. A collaborative effort involving obstetricians, neonatologists, pediatric surgeons, genetic counselors, and other specialists ensures a comprehensive evaluation and tailored counseling approach. Transparent and empathetic communication is crucial to ensure parents fully understand the nature and implications of the anomaly, empowering them to make informed decisions. Discussion of available treatment options, including surgical correction when applicable, provides parents with a clear understanding of potential interventions and their implications. It is fundamental to respect the autonomy of parents in decision‐making and to foster a collaborative and patient‐centered approach, aligning medical recommendations with the family's values and beliefs.

Neonatal counseling involves preparing parents for the birth of the baby, coordinating with neonatal intensive care units when necessary, and facilitating a smooth transition to postnatal care. Postnatal counseling ensures ongoing support as parents navigate the challenges of caring for a newborn with congenital anomalies. This may include addressing evolving medical needs, providing resources, and offering emotional support.

Overall, prenatal counseling is a dynamic and ongoing process that recognizes the unique needs of each family. By combining medical expertise with compassionate communication, healthcare professionals aim to optimize outcomes for both the newborn and the parents, promoting holistic care throughout the perinatal period.

## AUTHOR CONTRIBUTIONS

L.L. and E.B. designed and drafted the manuscript, critically reviewed the manuscript for important intellectual content, approved the final version to be published, and agreed to be responsible for all aspects of the work. C.R., A.B., M.P., P.L.C., F.G.S., G.Ch., G.Co., O.C., and A.L.M contributed to the design the work and draft of the manuscript. They also agree to be responsible for all aspects of the work. Given the multidisciplinary nature of counseling, each author has integrated their part of the work into the review. Each author meets the criteria for authorship.

## FUNDING INFORMATION

The present study did not receive any specific grants from funding agencies in the public, commercial, or not‐for‐profit sectors.

## CONFLICT OF INTEREST STATEMENT

The authors have no conflicts of interest.

## Data Availability

Research data are not shared.

## References

[1] Carlson LM , Vora NL . Prenatal diagnosis: screening and diagnostic tools. Obstet Gynecol Clin N Am. 2017;44(2):245‐256. doi:10.1016/j.ogc.2017.02.004 PMC5548328 28499534

[2] World Health Organization . Congenital disorders. Accessed March 2024. https://www.who.int/news‐room/fact‐sheets/detail/birth‐defects

[3] Euracat technical report. 2021 https://eu‐rd‐platform.jrc.ec.europa.eu/system/files/public/EUROCAT‐Statistical‐Monitoring‐Report‐2021.pdf

[4] Dolk H , Loane M , Teljeur C , et al. Detection and investigation of temporal clusters of congenital anomalies in Europe: seven years of experience of the EUROCAT surveillance system. Eur J Epidemiol. 2015;30(11):1153‐1164. doi:10.1007/s10654-015-0012-y 25840712 PMC4684832

[5] Lowry RB , Bedard T , Grevers X , et al. The Alberta congenital anomalies surveillance system: a 40‐year review with prevalence and trends for selected congenital anomalies, 1997‐2019. Health Promot Chronic Dis Prev Can. 2023;43(1):40‐48. doi:10.24095/hpcdp.43.1.04 36651885 PMC9894292

[6] Whitworth M , Bricker L , Mullan C . Ultrasound for fetal assessment in early pregnancy. Cochrane Database Syst Rev. 2010;4(4):CD007058. 10.1002/14651858.CD007058.pub2 PMC4084925 20393955

[7] Crane JP , LeFevre ML , Winborn RC , et al. A randomized trial of prenatal ultrasonographic screening: impact on the detection, management, and outcome of anomalous fetuses. The RADIUS study group. Am J Obstet Gynecol. 1994;171(2):392‐399. 8059817 10.1016/s0002-9378(94)70040-0

[8] Sileo FG , Finarelli A , Contu G , et al. Ultrasound screening for fetal anomalies in a single center: diagnostic performances twenty years after the Eurofetus study. J Matern Fetal Neonatal Med. 2022;35(25):6312‐6319. doi:10.1080/14767058.2021.1911994 33910476

[9] Grandjean H , Larroque D , Levi S . The performance of routine ultrasonographic screening of pregnancies in the Eurofetus study. Am J Obstet Gynecol. 1999;181(2):446‐454. 10454699 10.1016/s0002-9378(99)70577-6

[10] Glinianaia SV , Morris JK , Best KE , et al. Long‐term survival of children born with congenital anomalies: a systematic review and meta‐analysis of population‐based studies. PLoS Med. 2020;17(9):e1003356. doi:10.1371/journal.pmed.1003356 32986711 PMC7521740

[11] Marokakis S , Kasparian NA , Kennedy SE . Prenatal counselling for congenital anomalies: a systematic review. Prenat Diagn. 2016;36(7):662‐671. doi:10.1002/pd.4836 27150825

[12] Liao Y , Wen H , Ouyang S , et al. Routine first‐trimester ultrasound screening using a standardized anatomical protocol. Am J Obstet Gynecol. 2021;224(4):396.e1‐396.e15. doi:10.1016/j.ajog.2020.10.037 33127430

[13] Karim JN , Roberts NW , Salomon LJ , Papageorghiou AT . Systematic review of first‐trimester ultrasound screening for detection of fetal structural anomalies and factors that affect screening performance. Ultrasound Obstet Gynecol. 2017;50(4):429‐441. doi:10.1002/uog.17246 27546497

[14] Kowalczyk K , Bartnik‐Głaska M , Smyk M , et al. Comparative genomic hybridization to microarrays in fetuses with high‐risk prenatal indications: polish experience with 7400 pregnancies. Genes (Basel). 2022;13(4):690. doi:10.3390/genes13040690 35456496 PMC9032831

[15] McMullan DJ , Eberhardt RY , Rinck G , et al. Prenatal exome sequencing analysis in fetal structural anomalies detected by ultrasonography (PAGE): a cohort study. Lancet. 2019;393:747‐757. doi:10.1016/S0140-6736(18)31940-8 30712880 PMC6386638

[16] Serra G , Memo L , Coscia A , et al. Their respective scientific societies and Parents' associations. Recommendations for neonatologists and pediatricians working in first level birthing centers on the first communication of genetic disease and malformation syndrome diagnosis: consensus issued by 6 Italian scientific societies and 4 parents' associations. Ital J Pediatr. 2021;47(1):94. doi:10.1186/s13052-021-01044-1 33874990 PMC8054427

[17] Lakhoo K . Fetal counselling for surgical conditions. Early Hum Dev. 2012;88(1):9‐13. doi:10.1016/j.earlhumdev.2011.11.004 22142503

[18] Patel P , Farley J , Impey L , Lakhoo K . Evaluation of a fetomaternal–surgical clinic for prenatal counselling of surgical anomalies. Pediatr Surg Int. 2008;24:391‐394. 18256844 10.1007/s00383-008-2118-8

[19] Aite L , Trucchi A , Nahom A , Zaccara A , La Sala E , Bagolan P . Antenatal diagnosis of surgically correctable anomalies: effects of repeated consultations on parental anxiety. J Perinatol. 2003;23:652‐654. 14647162 10.1038/sj.jp.7210992

[20] Gallo M , Agostiniani R , Pintus R , Fanos V . The child with medical complexity. Ital J Pediatr. 2021;47(1):1. doi:10.1186/s13052-020-00935-z 33407754 PMC7788740

[21] Owens SN , Shorter JM . Pregnancy options counseling. Curr Opin Obstet Gynecol. 2022;34(6):386‐390. doi:10.1097/GCO.0000000000000823 36165041

[22] Carter BS , Parravicini E , Benini F , Lago P . Editorial: perinatal palliative care comes of age. Front Pediatr. 2021;9:709383. doi:10.3389/fped.2021.709383 34239852 PMC8257932

[23] Rusalen F , Cavicchiolo ME , Lago P , Salvadori S , Benini F . Perinatal palliative care: a dedicated care pathway. BMJ Support Palliat Care. 2021;11(3):329‐334. doi:10.1136/bmjspcare-2019-001849 31324614

[24] Benini F , Congedi S , Rusalen F , Cavicchiolo ME , Lago P . Barriers to perinatal palliative care consultation. Front Pediatr. 2020;8:590616. doi:10.3389/fped.2020.590616 33072680 PMC7536314

[25] Hjort‐Pedersen K , Olesen AW , Garne E , Toerring PM , Wu C , Sperling L . Parental information about the option to apply for pregnancy termination after the detection of a congenital abnormality and factors influencing parental decision‐making: a cohort study. BMC Pregnancy Childbirth. 2022;22(1):948. doi:10.1186/s12884-022-05255-0 36528557 PMC9759856

